# Ethnic differences in leptin and adiponectin levels between Greenlandic Inuit and Danish children

**DOI:** 10.3402/ijch.v72i0.21458

**Published:** 2013-08-07

**Authors:** Thor Munch-Andersen, Kaspar Sorensen, Niels-Jacob Aachmann-Andersen, Lise Aksglaede, Anders Juul, Jørn W. Helge

**Affiliations:** 1 Department of Biomedical Sciences, Center for Healthy Aging, University of Copenhagen, Denmark; 2 Department of Growth and Reproduction, Rigshospitalet, Faculty of Health and Medical Science, Copenhagen University Hospital, Denmark

**Keywords:** leptin, adiponectin, children, metabolic syndrome, ethnic differences, Inuit, leptin/adiponectin ratio

## Abstract

**Objective:**

In a recent study, we found that Greenlandic Inuit children had a more adverse metabolic profile than Danish children. Aerobic fitness and adiposity could only partly account for the differences. Therefore, we set out to evaluate and compare plasma leptin and adiponectin levels in Danish and Inuit children.

**Methods:**

In total, 187 Inuit and 132 Danish children (5.7–17.1 years) had examinations of anthropometrics, body fat content, pubertal staging, fasting blood and aerobic fitness.

**Results:**

Plasma leptin was higher in Danish boys [3,774 (4,741–3,005)] [pg/mL unadjusted geometric mean (95% CI)] compared to both northern [2,076 (2,525–1,706)] (p<0.001) and southern (2,515 (3,137–2,016)) (p<0.001) living Inuit boys and higher in Danish girls [6,988 (8,353–5,847)] compared to southern living Inuit girls [4,910 (6,370–3,785)] (p=0.021) and tended to be higher compared to northern living Inuit girls [5,131 (6,444–4,085)] (p=0.052). Plasma adiponectin was higher for both Danish boys [22,359 (2,573–19,428)] [ng/mL unadjusted geometric mean (95% CI)] and girls [26,609 (28,994–24,420)] compared to southern living Inuit boys [15,306 (18,406–12,728)] and girls [18,864 (22,640–15,717)] (both p<0.001), respectively. All differences remained after adjustment for body fat percentage (BF%), aerobic fitness, age and puberty. The leptin/adiponectin ratio was higher in Danish boys and tended to be higher in Danish girls compared to northern living Inuit boys and girls, respectively. These differences were eliminated after adjustment for BF%, aerobic fitness, age and puberty.

**Conclusions:**

In contrast to our hypothesis, plasma leptin was higher in Danish children despite a more healthy metabolic profile compared to Inuit children. As expected, plasma adiponectin was lowest in Inuit children with the most adverse metabolic profile.

The prevalence of metabolic morbidity differs across ethnic populations suggesting that some populations may be more genetically susceptible to lifestyle changes than others, for example Inuit, Pima Indians and Asia pacific populations (1–5). The results from multi-ethnic studies on genes involved in metabolism suggest the existence of ethnic variations, but investigations of risk genes for type II diabetes in different races have found no inter-ethnic variations (6–8).

In a recent study (9), we observed a more adverse metabolic profile in Greenlandic Inuit children compared to Danish children, indicating that the increase in metabolic morbidity observed in adult Greenlandic Inuit (5) may be traced to the juvenile Greenlandic Inuit population. The difference in metabolic risk profiles between Danish and Inuit children could only partly be explained by differences in adiposity and aerobic fitness level. Thus, to elaborate on our observations, we performed additional blood tests for ethnic differences in plasma leptin and adiponectin concentrations.

Leptin and adiponectin are secreted from adipose tissue and are linked to obesity, metabolic morbidity, insulin resistance and energy homeostasis (10,11). It has been reported in adults that differences across ethnic groups exist in adipokine levels and in their association to metabolic risk markers. These ethnic differences are proposed to provide at least some explanation for ethnic differences in insulin resistance (12,13).

Thus, we hypothesized that plasma leptin levels would be higher and plasma adiponectin levels lower in Inuit children with a more adverse metabolic profile than in Danish children.

## Methods

One hundred and thirty-two Danish children (8.5–16.1 years) and 187 Inuit children (5.7–17.1 years) participated. The Danish children were part of the Copenhagen Puberty study and recruited from schools near Copenhagen, Denmark. The participants and methods have been described previously (9,14–16). The Inuit children were recruited from public schools in Greenland. Before arrival, written material was distributed to the school and in the school we presented the projects to all classes individually. We aimed to include children from all age groups in order to get an equal distribution of children in the different puberty stages. From the capital in the south, Nuuk (55 boys and 47 girls), from Qaanaaq, the most northern located village (31 boys and 40 girls), and all school children from Siorapaluk, the most northern located all-year inhabited village-settlement in Greenland (12 boys and 2 girls). In Nuuk, children were recruited from the same school in the centre of the town, which according to teachers and local social workers is the area with the lowest socio-economic status. Thus, Inuit children were not randomly selected and thereafter invited to participate. There are approximately 1,900 children in 4 public schools in Nuuk (450–550 children in each). All schools have children aged 5–17 years. In Qaanaaq, there are approximately 160 children aged 5–17 years in one single school and in Siorapaluk, approximately 17 children aged 5–17 years in one school. We chose to recruit Inuit from the capital in the south hypothesizing that the influence of western lifestyle (type of diet and level of physical activity) would be most pronounced. In contrast, we hypothesized that adherence to a traditional lifestyle including consumption of marine diet would be most pronounced in the small, isolated and most northern located villages and settlements. In a study of 20 adult traditional living Inuit from Qaanaaq and the nearby settlements (17), we found almost complete adherence to traditional hunting and fishing and daily consumption of arctic marine foods. However, we have no quantitative measurements to support our hypothesis of differences in lifestyle between the children living in the north and the south.

All investigations in Greenland were carried out in August, in the north in 2007 and in Nuuk in 2008. Inuit origin was defined as having only one or no non-Inuit relative among the parents and grandparents. This was confirmed by asking the children in the presence of one or both their parents and, if necessary, with the help of an interpreter. The Danish participants were tested in 2008 from August to October. The same protocols, equipment, analytical methods and examiners were involved in testing both the Danish and the Inuit children. None of the children reported any medical conditions that could affect the measurements.

All children arrived in the morning after 10 hours of fasting. Blood samples were obtained and pubertal staging determined by a trained paediatrician, and anthropometrics and blood pressure were measured [systolic blood pressure (sysBP), diastolic blood pressure (diaBP)]. The children were subsequently offered a light meal, which was consumed at least 90 minutes before performing the maximal aerobic fitness test.

### Pubertal development

Pubertal development was evaluated according to Tanners Classification (18) by a trained paediatrician. Testicular volume was estimated by palpation using Praders Orchidometer. The date of last menstrual bleeding was recorded in post-menarche girls.

### Body composition

Body composition was evaluated by height, weight, body mass index (BMI), skin-fold thickness and bioelectrical impedance analysis (BIA) in all children (Holtain Ltd., Crymych, UK). In addition, the Danish children were whole body dual-energy X-ray absorptiometry (DEXA) scanned [Hologic CDR 1000/W densitometer (Hologic Inc., Bedford, MA, USA)] with estimations of fat-free mass (FFM) and fat mass (FM). Sex-specific formulas for estimation of FFM were developed based on the Danish children with valid data from both DEXA and BIA. From these data, body fat percentage (BF%) was calculated. Calculations and formulas have been described in detail previously (9). Skin-fold thickness was measured with a skin-fold calliper (Harpenden, West Sussex, UK) at the suprailiac, subscapular, triceps and biceps location. The sum of skin folds (SFsum) was calculated by adding all of the measurements.

### Blood samples

In the fasted state, venous blood was drawn from an antecubital vein and was subsequently centrifuged at 3,000 rpm for 10 minutes. Plasma was retrieved and stored at −20°C.

### Aerobic fitness

Maximal oxygen uptake (VO_2_max) was assessed as a measure of aerobic fitness using a progressive cycle ergometer test with an electronically braked cycle ergometer (Ergomedic 839; Monark, Varberg, Sweden), calibrated on every test day. Oxygen uptake was measured directly using an online pulmonary gas analyzer system (Quark CPET, Cosmed, Rome, Italy). Heart rate (HR) was recorded continuously throughout the test using a heart rate monitor (Polar Electro, Oulu, Finland). There were 2 different protocols used, depending on the age of the child. The work protocols and the criteria for a satisfactory maximal effort have been described previously (19,20).

### Analyses

All blood samples from the Inuit and Danish children were analyzed using the same methods, in the same laboratory. The blood and plasma samples from Greenland were stored and subsequently transported on dry ice to Copenhagen within a few days where they were stored at −20°C until analyzed.

Plasma adiponectin and leptin were measured using specific high-sensitive human enzyme-linked immunosorbent assay (ELISA) kits. The adiponectin assay (Millipore, Human ADIPONECTIN RIA-kit, St. Charles, MI, USA) had an intra-assay coefficient of variation (CV) of 4.9% and an inter-assay CV of 5.4% and detection limits of 1–200 ng/mL. The leptin assay (R&D Systems, Human Leptin Immunoassay, Minneapolis, MN, USA) had an intra-assay CV of 1.6% and an inter-assay CV of 3.4% and detection limits of 7.8–1,000 pg/mL.

Glycosylated haemoglobin (HbA1c), high-sensitive C-reactive protein (hsCRP), fasting insulin, fasting glucose, total cholesterol (TC), low-density lipoprotein-cholesterol (LDL-C), high-density lipoprotein-cholesterol (HDL-C), triglyceride (TG), Apolipoprotein A1 (ApoA), Apolipoprotein (ApoB) were determined by commercially available assays as previously described (9).

### Statistics

All statistical analyses were done using IBM SPSS 20.0 for Windows XP. Baseline characteristics are presented as mean±SD (Table I). Simple group comparisons were done using Student's *t*-tests for continuous variables and Fisher exact test for categorical data. Pearson correlations were used for simple correlations between the main outcome variables. Leptin and adiponectin levels as well as leptin/adiponectin ratio (L/P) were log-transformed to obtain approximate normal Gaussian distributions of the residuals as well as to obtain a residual variance that did not depend on the level. Differences in leptin, adiponectin and L/A between the Danish and the Inuit children were evaluated using general linear models with the respective adipokine level or L/A included as dependent variables, the 3 groups (Danish, southern living Inuit, northern living Inuit) and puberty (breast or genital stage 1–5) included as independent group variables and age, BF% and aerobic fitness levels included as continuous variables. Analyses were done separately for girls and boys. Adjustments for age and puberty were done in order to account for the uneven distribution of pubertal stages and the significant difference in mean age between the groups. Additional adjustments were done for adiposity and aerobic fitness in order to test if these factors could contribute to the differences in adipokine levels between the groups. Similarly, the associations between metabolic risk markers and adipokine levels and L/A were evaluated by general linear models with metabolic risk markers as a dependent variable and the respective adipokine levels as continuous variables. In order to evaluate differences in the association (regression slope) between the 3 different located groups, an interaction term between group location and adipokine levels was included.

**Table I:** Characteristics of southern and northern living Inuit and Danish boys and girls

	Boys			Girls		
		
	Danish, n=62	South, n=55	North, n=43	Danish, n=70	South, n=47	North, n=42
Pubertal stage (I–V) (%)	34/24/11/11/19	44/20/13/18/5	49/21/14/2/14	14/11/19/43/13	30/21/21/21/6	37/12/17/22/12
Age (years)	12.4±2.0* †	11.7±2.0	10.9±3.0	12.3±2.1* †	11.3±2.1	11.2±2.7
BF (%)	20.1±4.8†	19.6±5.5	18.2±4.3	23.2±4.1	24.1±4.6^‡^	22.2±4.0
Overweight/obese (%)	12.9	20.0	18.6	11.4	31.9* ‡	7.9
Aerobic fit (mL kg^−1^ min^−1^)	46.2±7.2†	46.7±6.0‡	49.4±6.0	40.1±5.7†	39.2±6.6‡	42.8±5.3

Classification of overweight and obesity were based on 2000 CDC Growth Chart. Results are mean±SD.

* p <0.05 (Danish vs. south);

† p<0.05 (Danish vs. north);

‡ p<0.05 (north vs. south).

### Ethics

The study was performed in accordance with the Declaration of Helsinki II. The study protocols were approved by the ethics committee of the capital region of Denmark (J.nr. KF 01 282214, KF 11 2006-2033) and the commission for scientific research in Greenland (J.nr 505-117). All children and their parents gave their informed written consent.

## Results

### Adipokines in relation to metabolic risk parameters, age and puberty

In all of the boys, leptin was positively correlated with BMI, SFsum, BF%, fasting insulin, hsCRP and negatively correlated with aerobic fitness level and apoA. In boys, adiponectin only correlated negatively with diaBP. In all of the girls, leptin was positively correlated with BMI, SFsum, BF%, fasting insulin, hsCRP, TG, diaBP and negatively correlated with aerobic fitness and apoA. In girls, adiponectin was negatively correlated with BMI, SFsum, BF%, HbA1c and positively correlated to aerobic fitness level and ApoA (all p<0.05).

In all of the boys, L/A was positively correlated with BMI, SFsum, BF%, fasting insulin, hsCRP, sysBP and diaBP and negatively correlated with aerobic fitness level (all p<0.012). In all of the girls, L/A ratio was positively correlated with BMI, SFsum, BF%, fasting insulin, hsCRP, sysBP and TG and negatively correlated with aerobic fitness level and ApoA (all p<0.023).

In all of the girls, the plasma leptin concentration and the L/A increased significantly with age (both p<0.001) and pubertal stage (both p<0.001) and the plasma adiponectin concentration decreased with age (p=0.007), but not with pubertal stage. In all of the boys, plasma leptin (but not adiponectin) levels showed a biphasic pattern during puberty with leptin levels increasing from genital stage 1 to 2 and decreasing from genital stage 2 to 5 (p=0.015). In all of the boys, the L/A tended to follow the same pattern as leptin during puberty (p=0.06). Age was not associated with plasma leptin, adiponectin or L/A in boys.

No significant interactions in the above correlation analysis (differences in regression slopes) were found between the 3 different groups for either boys or girls.

### Differences in adipokines between Danish, southern and northern Inuit children

Significant differences were found for leptin, adiponectin and L/A between the 3 groups (Table II and III).

**Table II:** Adipokines in southern and northern living Inuit and Danish boys

	Boys		
	
	Danish, n=62	South, n=55	North, n=43
Leptin, pg/mL	3,774	2,515	2,076
(CI)	(4,741–3,005)	(3,137–2,016)	(2,525–1,706)
p-value	<0.001*, <0.001†		
Adjusted p-value	0.046*, 0.001†		
Adiponectin, ng/mL	22,359	15,306	20,070
(CI)	(2,573–19,428)	(18,406–12,728)	(23,358–17,245)
p-value	<0.001*	0.035‡	
Adjusted p-value	<0.001*	0.041‡	
L/A ratio	0.16	0.16	0.10
(CI)	(0.21–0.13)	(0.22–0.12)	(0.14–0.08)
p-value	0.022†	0.027‡	
Adjusted p-value	0.046†	0.76‡	

Results are unadjusted geometric mean and 95% CI. Adjusted p-values represent differences after adjustment for body fat percentage, aerobic fitness, age and puberty.

* Danish vs. south;

† Danish vs. north;

‡ north vs. south.

**Table III:** Adipokines in southern and northern living Inuit and Danish girls

	Girls		
	
	Danish, n=70	South, n=47	North, n=42
Leptin, pg/mL	6,988	4,910	5,131
(CI)	(8,353–5,847)	(6,370–3,785)	(6,444–4,085)
p-value	0.021*, 0.052†		
Adjusted p-value	0.009*, 0.67†		
Adiponectin, ng/mL	26,609	18,864	29,496
(CI)	(28,994–24,420)	(22,640–15,717)	(34,952–24,891)
p-value	<0.001*	0.001‡	
Adjusted p-value	<0.001*	0.009‡	
L/A ratio	0.26	0.26	0.18
(CI)	(0.32–0.21)	(0.36–0.19)	(0.24–0.13)
p-value	0.054†		
Adjusted p-value	0.76†		

Results are unadjusted geometric means and 95% CI. Adjusted p-values represent differences after adjustment for body fat percentage, aerobic fitness, age and puberty.

* Danish vs. south;

† Danish vs. north;

‡ north vs. south.

Inuit children from the north had only Inuit parents and grandparents, whereas 3 girls and 14 boys from the south had 1 parent or 1 grandparent of Danish decent. Excluding these from the analysis did not change any of the findings.

## Discussion

This study is the first study comparing leptin and adiponectin levels in Inuit children with other ethnic groups of children. The primary finding in this study was the higher level of plasma leptin found in the more metabolically healthy Danish children compared to all Inuit boys and southern living Inuit girls. These results were in direct contrast to our hypothesis. As expected, the plasma adiponectin levels were highest in the Danish children.

Consistent results show that leptin and adiponectin are associated with adiposity and aerobic fitness and greater levels of leptin; lower levels of adiponectin are associated with metabolic morbidity and with modulating metabolic risk (10,21–25). Ethnic comparative studies of adults have shown higher levels of leptin and lower levels of adiponectin in the ethnic populations with the highest prevalence of metabolic syndrome, even when degree of adiposity and other confounders is accounted for (12,13,25). In children, higher mean levels of leptin as well as greater relative changes in metabolic risk markers for given changes in leptin levels have been demonstrated in South Asian children compared with Caucasian and African-Caribbean children (26). A study of 74 African-American and Caucasian pre-pubertal children found no ethnic differences in leptin levels when adjusting for differences in body composition measured by DEXA (27). In contrast, a study comparing Italian and west African children aged 6–10 years found that at all levels of BMI, the Italian children showed a greater level of leptin than the Gambian children (28). However, the use of BMI in growing children and in inter-ethnic studies has its limitations. In contrast to our *a priori* hypothesis, we found higher leptin levels in Danish children compared to Inuit children who had a more adverse metabolic profile and higher prevalence of being overweight. These differences remained significant when differences in adiposity, aerobic fitness, age distribution and pubertal development between the groups were accounted for. In addition, the associations between leptin levels and metabolic risk markers did not differ between Danish and Inuit children, implying that Danish and Inuit children are equally metabolically sensitive to changes in leptin levels.

Plasma leptin in the non-disease/non-obese state is involved in feeding regulation and thermogenesis (29,30). Studies have indicated that variations in the leptin receptor gene exist across ethnic populations, but the relationship between these variations and obesity and metabolic morbidity is inconclusive (31). Observations of human morphology in relation to different climates have shown significantly more heat conserving body anthropometrics in populations living in cold environments (32). These observations are supported by studies showing that climate exerts a selective pressure on candidate genes for metabolic regulation and associated disorders, including the leptin receptor gene (7). Thus, the lower levels of leptin in Inuit children in the present study compared to Danish children, when normalized for body fat may reflect an evolutionary drive towards a more heat conserving body composition. Thus, the difference in leptin may primarily represent normal physiological ethnic variation as opposed to adverse pathometabolic processes.

Adiponectin levels were lowest in the southern living children compared to the northern living Inuit and the Danish children. The Inuit children, especially those from the south and most pronounced in girls, had the most adverse metabolic profile (9). Adiponectin is negatively associated with several known associates and modulators of metabolic morbidity in adults and children (10). Thus, these findings may more likely be interpreted as being part of an ongoing pathometabolic process. However, in the present study adiponectin levels were only associated with several metabolic risk factors in girls. In boys, adiponectin were only negatively correlated with diaBP.

The L/A has been observed to be asociated with markers of metabolic risk and to provide a precise estimate of insulin resistance both in individuals with and without hyperclycemia (33–36). In this study, L/A correlated well with several metabolic risk factors. The highest L/A was found in the Danish children with the healthiest metabolic profile. A recent study observed a positive effect on L/A of omega-3 polyunsaturated fatty acids in patients with stable coronary artery disease (37). The lowest L/A was found in the northern living Inuit children where adherence to a traditional Arctic lifestyle and marine diet is hypothesized to be more complete than in Nuuk in the south. However, in contrast to the differences in leptin and adiponectin, the differences in L/A were eliminated when acconting for the slightly higher aerobic fitness level and lower BF% in the north and the differences in age and pubertal development.

## Strengths and limitations

Diet, socio-economic status, drinking and smoking habits may influence the results. The recruitment pattern in the area with the lowest socio-economic status in Nuuk could mean that the population studied in Nuuk may not be representative of the Inuit children living in the south. In the north, almost half of the children population in the area participated. Interestingly, a recent study did not report adverse effect of urbanization in adults in Greenland (38). Several metabolic risk factors fluctuate significantly during puberty and a major strength of our study is the thorough pubertal classification performed by trained paediatricians. The equations used to estimate BF% were validated by DEXA in Danish children only. The single-frequency BIA method is dependent on the diameter and length of the body cylinders (extremities and trunk). Ethnic differences in anthropometrics may have resulted in incorrect estimates of FFM and FM in the Inuit. In addition, residual confounding in the adjustment for aerobic fitness and fatness should also be taken into consideration.

In conclusion, in contrast to our *a priori*. Hypothesis, we found lower levels of circulating leptin in Inuit children compared to Danish children despite Inuit children having a more unfavourable metabolic profile, including higher prevalence of overweight and obesity. The differences decreased but remained significant after adjustment for aerobic fitness level, adiposity, age and puberty. The lower plasma leptin levels in the Inuit children may represent genetic variation between Inuit and Danish children. As hypothezied, lower levels of adiponectin were found in the Inuit children with the most adverse metabolic profile. This may be directly related to the more adverse metabolic risk profile in Inuit children.
